# Modified Milk for Young Animals1Read at the annual meeting of the Pennsylvania State Veterinary Medical Association, March, 1898.

**Published:** 1898-04

**Authors:** C. J. Marshall

**Affiliations:** Instructor in Veterinary Medicine, Veterinary Department, University of Pennsylvania


### MODIFIED MILK FOR YOUNG ANIMALS.[1]

By C. J. Marshall, V.M.D.,
INSTRUCTOR IN VETERINARY MEDICINE, VETERINARY DEPARTMENT, UNIVERSITY OF PENNSYLVANIA.

Milk from all mammalians in its pure state contains the following ingredients: water, casein, albumin, fat, sugar, and salts—the salts being made up of sodium, potassium, chlorine, calcium, phosphoric acid, sulphuric acid, ferric oxide, and a trace of silica. Nature has arranged the proportion of these ingredients in the milk of the different species of animals in the manner best adapted to the need of the young of each particular species. The arrangement is interesting to us, for the reason that it often becomes necessary to substitute the milk of one species for that of another in feeding young animals.

We have, perhaps, all experienced trouble in trying to raise lambs, colts, and puppies on cow's milk. When troubles arise, fats are blamed, and milk less rich in fat is substituted, and perhaps sugar is added, not so much for its physical action as to tempt the animal's appetite. Work in this line is usually done in an empirical manner, and frequently results in the death of the animal. The medical profession have recognized this fact in regulating the feeding of babies. The specialist on children's diseases at the present time must be thoroughly familiar with all the constituents of milk, and be able when necessary to direct how the arrangement of these ingredients in cow's milk may be changed in order to make it as near like the mother's milk as possible. This is very convenient where the prescription or directions can be sent to a laboratory, where every facility is at hand for carrying on this kind of work.

Unfortunately, our clients do not live near enough to such conveniences to make it practicable for us. Yet, with a little study, and calculation from the following table, much good might be accomplished on our part in directing the modification of milk so that it will be suitable for the different species for which we have to prescribe.

This table was prepared by König as a result of his own experiments, combined with those of Pfeiffer, Biehl, and Fleischman.

[1] Read at the annual meeting of the Pennsylvania State Veterinary Medical Association, March, 1898.

This table differs slightly from the one found in Smith's *Physiology*, but is more recent:

| | Water. | Casein. | Albumen. | Fat. | Sugar. | Salts. |
|---|---|---|---|---|---|---|
| Woman . . . | 87.2 | 0.59 | 1.23 | 3.94 | 6.23 | .45 |
| Cow . . . . | 87.24 | 2.88 | .53 | 3.65 | 4.81 | .70 |
| Goat . . . . | 87.33 | 3.01 | .51 | 3.94 | 4.39 | .82 |
| Sheep . . . . | 81.31 | 5.28 | 1.03 | 6.83 | 4.73 | .82 |
| Mare . . . . | 91. | 1.32 | .67 | 1.18 | 5.31 | .43 |
| Ass . . . . . | 89.64 | .67 | 1.55 | 1.64 | 5.99 | .51 |
| Llama . . . . | 86.55 | 3. | .90 | 3.15 | 5.06 | .80 |
| Camel . . . . | 86.57 | . . | . . | 3.07 | 5.59 | .77 |
| Bitch . . . . | 75.44 | 6.10 | 5.05 | 9.57 | 3.09 | .73 |

The important food-constituents are found in the proteids, the fats, and the sugars. It is not only necessary that these constituents should exist in the milk, but they should be present in nearly the same proportion found in milk from the mother of the young animal upon which we desire to practice artificial feeding. These constituents should resemble those of the mother's milk both in their chemical properties and in their behavior to the digestive fluids. In infant-feeding it has been found useless to add substances foreign to the mother's milk, as starch, for instance. For artificial feeding, cow's milk is the most convenient substitute. We find it contains all the constituents, but not in the right proportion.

In order to modify cow's milk intelligently we must consider to what extent these constituents exist in the different ages and forms of cow's milk. As it comes from the cow, milk usually shows a double reaction, both alkaline and acid. But on standing a short time it becomes acid, due to lactic-acid fermentation. As a rule, it is said that fresh milk from herbivorous animals is alkaline in reaction, while that from the carnivora is acid.

If cow's milk that shows 4 per cent. fat when fresh is allowed to stand six hours in ice-water, cream will rise by gravitation. This cream, if removed and tested, will show 12 per cent. fat, while the under-milk, or skim-milk, will yield 2 per cent. of fat. If the same milk were allowed to stand in ice-water for twelve hours, 16 per cent. of cream would be obtained, which cream is the richest in fat that can be obtained by the gravity method. If the separator be used, it is possible to obtain cream containing a maximum of 48 per cent. fat, with but a slight trace of fat in the separator-milk. By either method the proportion of proteids and sugars is but slightly altered. 32 per cent. cream contains 3.40 per cent. sugar and 2.90 per cent. proteids. 16 per cent. cream contains

4.20 per cent. sugar and 3.60 per cent. proteids. 8 per cent. cream contains 4.40 per cent. sugar and 3.90 per cent. proteids. We see by this that as the percentage of cream decreases the percentage of sugar and proteids increases, and milk minus the fats is still rich in proteids and sugars.

The inorganic salts in milk are nearly constant, and so far no attempt has been made to modify their proportions. The proteids and sugar in milk are of just as much consequence as the fats, and perhaps more. In feeding children it is requisite that the percentage of proteids in the milk as modified approximate the standard to within one-fourth of 1 per cent. If we desire to raise the percentage of proteids above the value found in cow's milk, it becomes necessary to get it from some other source. The white of an egg has been found to answer the purpose, and can be considered as 100 per cent. albumin. When too low in sugar, milk-sugar is found the best substitute. If any or all of these constituents exist in too high a percentage, water may be added, knowing, of course, that adding half water will reduce the constituents by half.

Gravity-skimmed milk, which contains 2 per cent. of fat is too rich in fats for a colt, while gravity cream, which is about 10 per cent. fat, is very little too rich in fats for a puppy.

We must also consider the age of the infant for which we are prescribing. In human practice it has been found that for the first three days a child should have no proteids or fats in artificial feeding, hence a 5 per cent. solution of sugar in water is used. For the next week the sugar is increased, and six-tenths of 1 per cent. of proteids are added. At from six to nine months old the sugar is increased to 7 per cent. Then it is gradually decreased till the age of about eighteen months, when whole milk is used. The percentage of fats and proteids is not prescribed higher than 4 per cent. So far no substitute has been found for colostrum. It is known that this milk is rich in broken-down epithelial cells. A solution of sugar in water is found to be its best substitute.

You might wonder what the symptoms are when the fats, sugar, and proteids are fed in improper proportions. Much can be learned on this subject. To Rotch belongs the chief honor of adapting a scientific system of using modified milk. It has been used quite extensively in the larger cities for private feeding, and in children's hospitals.

The Walker-Gordon Laboratory Company, which have laboratories in several of the principal cities of the United States, is the only firm that has made extensive practical application of a truly scientific method of producing modified milk. It has been observed in feeding children that the gain in weight is apt to be slow when a deficiency of sugar is used. The excess of sugar causes the most trouble, which is usually indicated by frequent colics; thin, green, very acid stools; eructation of gases from the stomach, and regurgitation of small quantities of food. An excess of fats is indicated by regurgitation of small quantities of sour food an hour or two after feeding; frequent passages from the bowels, which are quite normal in appearance, but sometimes contain small lumps resembling casein, but which really are masses of fat. It rarely causes colic. A deficiency in fats usually results in constipation with dry, hard stools. It has not been found advisable to increase the fats above normal to overcome this trouble. Frequent colics, curds in the stools, diarrhœa, more often constipation, are usually indications of an excess of proteids; while a deficiency in proteids interferes with the growth of the infant.

A little ingenuity and knowledge of mathematics is required to change the ingredients in cow's milk so that it will be theoretically and practically similar in composition to the milk which we desire to imitate. We will note the changes necessary to modify cow's milk so that it will be suitable for a colt, lamb, or puppy :

| | Cow. | Sheep. | Bitch. | Mare. |
|---|---|---|---|---|
| Proteids . . . . . . . . . . . | 3.41 | 6.31 | 11.15 | 1.99 |
| Fats . . . . . . . . . . . . | 3.65 | 6.83 | 9.57 | 1.18 |
| Sugars . . . . . . . . . . . | 4.81 | 4.73 | 3.09 | 5.31 |

The *modus operandi* of modifying milk can be best illustrated by the solution of a few practical problems. Suppose it is required to modify skim-milk so that it will be suitable for a lamb. We have available for this purpose 16 per cent. cream, skim-milk, sugar of milk, egg-albumin, and water. We will first make the calculation for determination of the fats. Sheep's milk should contain 6.83 per cent. fat. Suppose we wish to prepare forty ounces of modified milk at one time. It should contain 6.83 per cent. of 40 ounces or 2.73 ounces of fat. How can we mix 2 per cent. skim-milk and 16 per cent. cream so that the mixture will contain 2.73 ounces of fat? Let $x =$ quantity of skin-milk, and $40 - x =$ quantity of 16 per cent. cream.

The $x$ ounces of skim-milk will contain $\frac{2x}{100}$ ounces of fat, and

$40 - x$ ounces of cream will contain $\frac{16\ (40 - x)}{100}$ ounces of fat, and together they will contain the sum of $\frac{2x}{100} - \frac{16(40-x)}{100} =$ 2.73.

$$2x - 640 - 16x = 273$$
$$14x = 367$$
$$x = 26\tfrac{3}{14}$$

So if we mix $26\frac{3}{14}$ ounces of skim-milk containing 2 per cent. of fat and $13\frac{11}{14}$ ounces of cream containing 16 per cent. of fat, we will have 40 ounces of mixture which will contain the same percentage of fat as does sheep's milk. Sheep's milk should contain 6.31 per cent. of proteids. The 40 ounces must contain 6.31 per cent. of 40 ounces, or 2.52 ounces of proteids.

Skim-milk contains about 4 per cent. proteids; so $26\frac{3}{14}$ ounces would contain 4 per cent. of $26\frac{3}{14}$ ounces of 1.05 ounces of proteids. 16 per cent. cream contains 3.6 per cent. proteids, then $13\frac{11}{14}$ ounces of cream would contain 3.6 per cent. of $13\frac{11}{14}$ ounces, or 1.50 ounces of proteids. We have then added the sum of 1.05 and .50 or 1.55 ounces of proteids. We must then add the difference between 2.52 and 1.55 ounces or .97 of an ounce of proteids, which is practically one ounce. The white of an egg weighs about one ounce, so we can add the white of one egg. The sugar is already within 0.1 per cent. correct, which is near enough. Our prescription will then read thus: ℞.—Cream, 16 per cent. fat, 14 ounces; skim-milk, 2 per cent. fat, 26 ounces; white of egg, one ounce. M. Sig.—Warm and feed as required.

Another example: We will consider modified milk suitable for a colt. We will use separator-milk, because skim-milk is already too rich in fats, and add 16 per cent. cream. In this case our prescription will read: For colt. ℞.—Cream, 16 per cent. fat, 3 ounces: separator-milk, 20 ounces; water, q. s. ad., 40 ounces; milk sugar, 1.2 ounces. M. Sig.—Use as directed. For puppy: ℞.—Cream, 16 per cent., 24 ounces; white of egg, 3 ounces; separator-milk, q. s. ad., 40 ounces. M. Sig.—Warm to blood-heat and feed as required.

In the last prescription the percentage of sugars is .20 per cent. too high, but this variation is permissible.

---

London (England) and Bremen (Germany) are still large buyers of horses in American markets, and Chicago in one week in March received and sold over 13,000 head.

